# The Impact of Low Latency Satellite Sounder Observations on Local Severe Storm Forecasts in Regional NWP

**DOI:** 10.3390/s20030650

**Published:** 2020-01-24

**Authors:** Pei Wang, Jun Li, Timothy J. Schmit

**Affiliations:** 1 Cooperative Institute for Meteorological Satellite Studies (CIMSS), University of Wisconsin–Madison, Madison, WI 53706, USA; pei.wang@ssec.wisc.edu; 2 ASPB/CoRP, Center for Satellite Applications and Research, NOAA, Madison, WI 20740, USA; tims@ssec.wisc.edu

**Keywords:** data latency, satellite data assimilation, local severe storm, regional numerical weather prediction

## Abstract

The forecasts of local severe storms (LSS) are highly dependent on how well the pre-convection environment is characterized in the numerical weather prediction (NWP) model analysis. The usefulness of the forecasts is highly dependent on how frequently the forecast is updated. Therefore, the data latency is critical for assimilation into regional NWP models for it to be able to assimilate more data within the data cut-off window. These low latency data can be obtained through direct broadcast sites and direct receiving systems. Observing system experiments (OSE) were performed to study the impact of data latency on the LSS forecasts. The experiments assimilated all existing observations including conventional data (from the global telecommunication system, GTS) and satellite sounder radiance data (AMSU-A (The Advanced Microwave Sounding Unit-A), ATMS (Advanced Technology Microwave Sounder), CrIS (Cross-track Infrared Sounder), and IASI (Infrared Atmospheric Sounding Interferometer)). They were carried out in a nested domain with a horizontal resolution of 9 km and 3 km in the weather research and forecasting (WRF) model. The forecast quality scores of the LSS precipitation forecasts were calculated and compared with different data cut-off widows to evaluate the impact of data latency. The results showed that low latency can lead to an improved and positive impact on precipitation and other forecasts, which indicates the potential application of LEO direct broadcast (DB) data in a high-resolution regional NWP for LSS forecasts.

## 1. Introduction

The advanced infrared (IR) and microwave (MW) sounders [1] onboard the current polar orbiting satellites (i.e., Joint Polar Satellite System (JPSS) series, Metop series, and FY-3 series) are equipped with atmospheric sounding capability in the pre-convection environment. This is important for improving high impact weather (HIW) event forecasts through assimilating the observations into numerical weather prediction (NWP) models [2,3,4]. The usefulness of the forecast is dependent on how frequently the forecast is updated. The importance of data latency was discussed and indicated that the improved data latency, more frequent data refresh, and enhanced data coverage from satellites facilitate more rapid updates to regional and mesoscale weather forecast models [5]. For example, the Rapid Refresh (RAP) is the continental-scale National Oceanic and Atmospheric Administration (NOAA) hourly-updated assimilation/modeling system that is operational at the National Centers for Environmental Prediction (NCEP). RAP is complemented by the higher-resolution, 3 km high-resolution rapid refresh (HRRR) model [6]. The RAP/HRRR provides hourly updated regional forecasts for high impact weather (HIW) events and the low latency observations are important for timely ingesting into the model. These low latency data from polar orbiting satellites can be obtained through direct broadcast (DB) sites [7].

The new generation of geostationary satellites such as the FengYun-4 (FY4) series [8], Geostationary Operational Environmental Satellite (GOES-R series) [9,10], and Himarari-8/-9 [11], with high spatial and temporal resolutions are capable of monitoring moisture changes in pre-convection. In addition, low latency can be obtained by receiving the geostationary rebroadcast (GRB) Level 1B (L1B) data. For example, the GOES-16 Advanced Baseline Imager (ABI) observes the full disk every 10 min, the Contiguous U.S. (CONUS) every five minutes, and a mesoscale domain every one minute, however, the current GOES-R series only has limited sounding capability in the pre-convection environment, especially with respect to monitoring vertical profiles.

Assimilating the low latency data from low earth orbit (LEO) and geostationary (GEO) into high resolution regional NWP models demonstrates a process for improving rapidly changing weather forecasts. Impact studies have been conducted through observing system experiments (OSEs) on the assimilation of radiances in regional NWP models. The focus has been on the impact of latency from polar orbiting satellite sounder data due to the current limited sounding capability from GEO orbit, as above-mentioned. In general, there is a trade-off between the number of observations and latency. Low latency ensures that observations are closer to the model analysis time, which is beneficial to NWP, but may result in fewer observations available for the model. In this study, all available observations including conventional data and satellite data were tested within different data latency schemes in the regional NWP model for LSS case simulation. The equitable threat score (ETS)/false alarm rate (FAR)/probability of detection (POD) scores of the LSS precipitation forecasts were calculated and compared with different data cut-off widows to evaluate the impact of data latency.

The rest of this article is organized as follows. Section 2 describes the datasets and methodologies used, Section 3 describes the experimental design, Section 4 provides the impact analysis on LSS forecasts, and the conclusions are summarized in Section 5.

## 2. Methodologies and Data

### 2.1. Data Latency

The current polar orbiting satellites such as from Suomi-NPP, JPSS, and Metop-A/B have atmospheric sounding capability in the pre-convection environment and low latency can be obtained through DB sites over the CONUS and adjacent regions. DB is the most rapid way to obtain the satellite observations from the sensor to the ground processing system. According to the NOAA Space Platform Requirements Working Group (SPRWG), which represents user needs, data latency is defined as the time it takes for the sensor to make the observation until the time the observation or product is available to the primary NOAA users (e.g., National Weather Service (NWS) forecasters or NCEP), so making the observation or product available to NOAA users must therefore include the data processing time. The processing time is dependent on the observation or product and can be a substantial fraction of the total latency [12]. For example, when converting the satellite observations to the standard BUFR (Binary Universal Form for the Representation of meteorological data) format or to the retrieval products for NWP models, satellite data latency can range from minutes to hours. This data latency directly affects the data assimilation and the NWP models.

In this study, the satellite data latency impacts were tested in an LSS study in regional NWP. To increase the latency impacts on the forecast results, the latency time was set as follows: 3-h, 2-h, 1-h, and no latency. A three-dimensional variational data assimilation (3Dvar) method was used for all of the experiments, with a 6-h assimilation window at the analysis time. Therefore, the 3-h latency represents the data starting at 3-h ahead of the analysis time to the analysis time; the 2-h latency represents the data starting at 3-h ahead of the analysis time to 1-h after the analysis time; the 1-h latency represents the data starting at 3-h ahead of the analysis time to 2-h after the analysis time; and no latency represents the data starting at 3-h ahead of the analysis time to 3-h after the analysis time. The 3-h latency was the largest latency with the smallest amount of satellite data, and the no latency condition provided the largest amount of satellite data during the 6-h assimilation window. The no latency condition is the ideal condition since it provides the most information at the time of analysis.

### 2.2. Satellite Sounder Observations Used in OSE (Binary Universal Form for the Representation of Meteorological Data) Impact Studies

**Figure 1:**
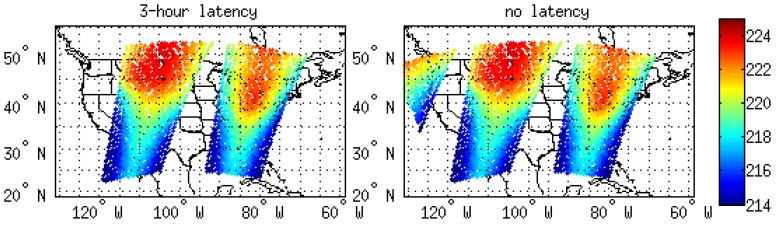
Channel 8 of AMSU-A onboard Metop-B data coverage for the 3-h latency and 2-h latency at 1800 UTC 23 June, 2018.

**Figure 2:**
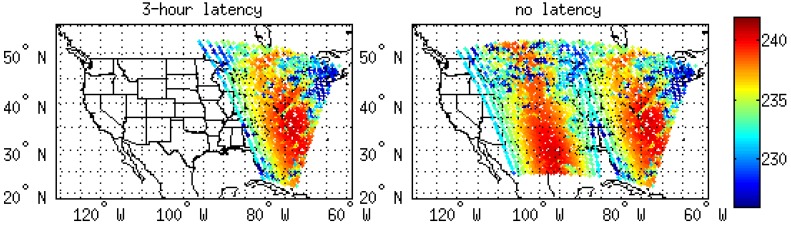
Channel 96 of CrIS data coverage for the 2-h latency and 1-h latency at 1800 UTC 23 June, 2018.

Based on the required latency conditions of 3-h, 2-h, 1-h, and no latency, the original 6-hourly observations for both the conventional data and satellite observations were divided into every 3-h, 4-h, and 5-h. The 3-h data translate to a 3-h latency, 4-h data translate to a 2-h latency, and the 5-h data translate to a 1-h latency (Table 1). If all of the 6-hourly data are used, then there is no latency, which is the best possible condition because it provides all of the information from the observation data in the assimilation window. For example, the data distribution of the Advanced Microwave Sounder Unit (AMSU)-A onboard Metop-B is shown in Figure 1, with an assimilation time of 1800 UTC on 23 June 2018. There is an additional granule of data from the 2-h latency that can be used in our domain and compared to the data from the 3-h latency. It indicates that the observation time of this extra granule of data occurs between the analysis time and 1-h post-analysis. These observations are not available for the 3-h latency experiment, but they can be assimilated for the 2-h latency experiment. Figure 2 provides the data distribution for Channel 96 of the Cross-track Infrared Sounder (CrIS) for both the 2-h and 1-h latency. In the 2-h latency, there are only a few observations covered between 90°W and 70°W longitude. In the 1-h latency, the data coverage expands from 120°W to 70°W longitude. There are more observations than can be used in the model domain for the 1-h latency, therefore, the assimilation of the extra observations would further affect the forecast results.

Observations from selected channels of AMSU-A, Advanced Technology Microwave Sounder (ATMS), CrIS, and Infrared Atmospheric Sounding Interferometer (IASI) [1] are listed in Table 2, showing the four different data latency experiments at 1800 UTC on 23 June 2018. The increasing rate of observations between the 3-h latency and no latency conditions were calculated and are shown in the last column of Table 2. The observations of AMSU-A onboard NOAA-15, NOAA-18, Metop-A, and IASI onboard Metop-A were consistent across the four different latency times, which indicates that there were no differences for these channels in the data coverage in our domain. The observations from Channel 6 of AMSU-A onboard Metop-B and Channel 110 of IASI onboard Metop-B increased approximately 10% from the 3-h latency to the no latency condition. The observations of ATMS and CrIS onboard Suomi-NPP showed an increase of greater than 50%. For AMSU-A onboard NOAA-19, there was no data coverage in the domain from the 3-h latency. The AMSU-A onboard NOAA-19 data were available since 2-h latency, so the increasing rate of it was 100%. In general, there were differences in the data coverage from the 3-h latency to 2-h latency and from the 2-h latency to 1-h latency. The difference in data coverage for the selected channels between the 1-h latency and no latency was marginally small. However, the different data coverage for other channels and conventional data together affected the forecast results of the 1-h latency and no latency experiments, respectively.

## 3. Experimental Designs for Impact Studies

### 3.1. Case Description

**Figure 3:**
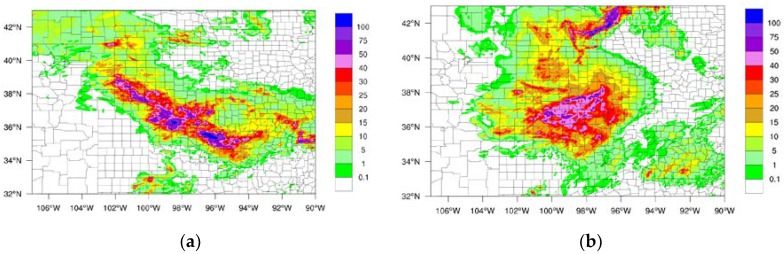
The 18-h accumulated precipitation (unit: mm) from 0000 UTC 24 June to 1800 UTC 24 June, 2018 (Case I) (**a**), and the 12-h accumulated precipitation from 0000 UTC 25 June to 1200 UTC 25 June, 2018 (Case II) (**b**) based on Stage-IV observations.

Two typical storm cases were selected in order to evaluate the data latency impact on the LSS. Stage IV precipitation dataset from NCEP as well as GOES-16 water vapor channel brightness temperatures (BTs) were used to detect the LSS case. Based on Stage IV and GOES-16 brightness temperatures, one LSS case ran from 000 UTC on 24 June to 1800 UTC on 24 June, 2018 (Case I), and another LSS case was from 0000 UTC on 25 June to 1200 UTC on 25 June 2018 (Case II). The 18-h accumulated precipitation for Case I is plotted in Figure 3a, and the 12-h accumulated precipitation for Case II is plotted in Figure 3b. For Case I, the LSS started between Colorado and Kansas, and then moved in a southeasterly direction to Oklahoma. The 18-h accumulated precipitation was greater than 100 mm from northwest Kansas to southeast Oklahoma. For Case II, the LSS covered Colorado, Kansas, and Oklahoma all together. The maximum 12-h accumulated precipitation was over 40 mm, and at very few regions was the precipitation over 50 mm.

### 3.2. Stage IV Dataset

The Stage IV dataset was used as the “truth” for verifying the precipitation forecast. The Stage IV analysis was based on the multi-sensor hourly/6-h “Stage III” analyses (on local 4 km polar-stereographic grids) produced by the 12 River Forecast Centers (RFCs) in CONUS [13]. NCEP mosaics the Stage III into a national product as the Stage IV dataset. It can be found in hourly, 6-hourly, and 24-hourly accumulated precipitation analyses. Furthermore, the NCEP Stage IV also includes the manual quality control performed on the Stage III data at the RFCs [14]. The Stage IV data are very useful for studies with high spatial resolution (4 km) data, and it is widely used for the study of quantitative precipitation forecasts [15,16]. Note that the maps of precipitation are generated by NCEP using a mosaicking technique that combines data from the 12 RFCs in the CONUS, which would bring bias to the data. Based on [17], the biases exist in the algorithms used by the RFCs as well as the operational processing at the radar site.

### 3.3. Data Assimilation System

The Developmental Testbed Center (DTC), supported the Community Gridpoint Statistical Interpolation (GSI) system, was used as the data assimilation system, which has the capability of assimilating nearly all of the existing observations including those from radiosondes, aircraft, microwave and infrared sounders, and radar. It is primarily a 3-Dvar system, and also has the option to be used as a hybrid data assimilation system [18]. The hybrid data assimilation in a GSI system is an ensemble Kalman filter-variational hybrid data assimilation system. It was developed collaboratively by NOAA, the National Aeronautics and Space Administration (NASA), and the National Center for Atmospheric Research (NCAR) for operational use. DTC provided the data assimilation community version along with the support for research study and some real-time models. Due to the limited resources in generating the ensemble members for hybrid assimilation in regional models, the 3-Dvar method was selected for use in this study. The satellite bias correction method uses the enhanced bias correction method [19], which is updated at every time step. The background and observation error covariance were from the North American Mesoscale Forecast System (NAM). The Community Radiative Transfer Model (CRTM) was used to assimilate the satellite radiances [20,21,22]. The CRTM version 2.2.3 coefficient was used for satellite simulation.

**Figure 4:**
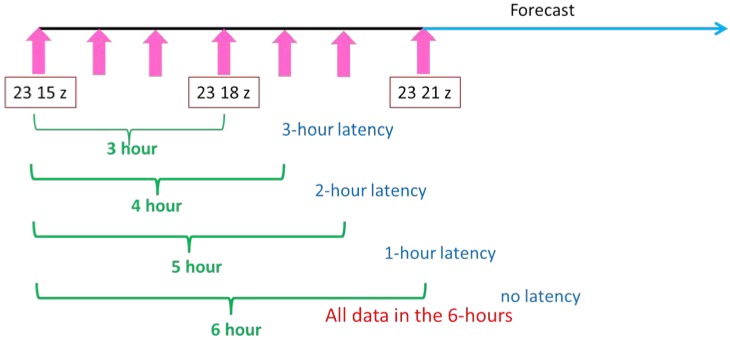
The flow chart of the assimilation and forecast experiments. The assimilation time was 1800 UTC 23 June. The green hours represent the available data time, and the blue represents the latency time.

Since the LSS Case I ran from 0000 UTC on 24 June to 1800 UTC on 24 June, 2018, the experiments started 6-h earlier, which was 1800 UTC on 23 June to 1800 UTC on 24 June, 2018. The assimilation time was 1800 UTC 23 June, and was followed by the forecasts from 0000 UTC on 24 June to 1800 UTC on 24 June, 2018. The 3-h latency represents the data only available from 1500 UTC 23 June to 1800 UTC 23 June; the 2-h latency represents the data available from 1500 UTC 23 June to 1900 UTC 23 June; and the 1-h latency represents the data available from 1500 UTC 23 June to 2000 UTC 23 June (Table 1). The experimental design is shown in Figure 4. Four experiments were conducted to simulate the LSS case with 3-h, 2-h, 1-h, and no latency. The assimilated data included AMSU-A data onboard NOAA-15, NOAA-18, NOAA-19, Metop-A and Metop-B; ATMS and CrIS data onboard SNPP; and IASI data onboard Metop-A and Metop-B.

LSS Case II was from 0000 UTC on 25 June to 1200 UTC on 25 June, 2018, where the experiments started from 0000 UTC 24 June, followed by a 36-h forecast. The 3-h latency represents the data from 2100 UTC 23 June to 0000 UTC 24 June; 2-h latency contains the data from 2100 UTC 23 June to 0100 UTC 24 June; 1-h latency contains the data from 2100 UTC 23 June to 0200 UTC 24 June; and no latency contains the data from 2100 UTC 23 June to 0300 UTC 24 June. All the experimental designs and the data assimilation schemes were the same as Case I.

### 3.4. WRF-ARW Regional NWP Model

The Advanced Research WRF (WRF-ARW) v 3.6.1 was used as the regional NWP model. WRF-ARW was developed by NCAR and is broadly used in both research studies and regional operational centers. The horizontal resolution of the regional model was 9 km and 3 km nested domains. The vertical layers were 50 layers from the surface to 10 hPa. The calculation time step was every 20 s. This model setting is an emulation of RAP/HRRR. The NCEP GDAS/FNL 0.25-degree data were used as the initial and boundary conditions for the regional model simulation. The cumulus schemes were not required for the WRF-ARW model since the horizontal resolutions of the models were less than 10 km. The Thompson aerosol-aware microphysics scheme was used to simulate the LSS precipitation. The RRTMG radiation scheme was used to calculate the longwave and shortwave radiation. The Yonesei University Scheme (YUS) was used as the planetary boundary layer (PBL) scheme. The pattern of the simulated rainfall was similar to the observations when using these physical schemes, however, the simulated precipitation was stronger than the observations, which was due to the 3 km high resolution of the inner, nested domain.

## 4. Results and Analysis

### 4.1. Impact of Low Latency Satellite Sounder Observations on Precipitation Forecasts

**Figure 5:**
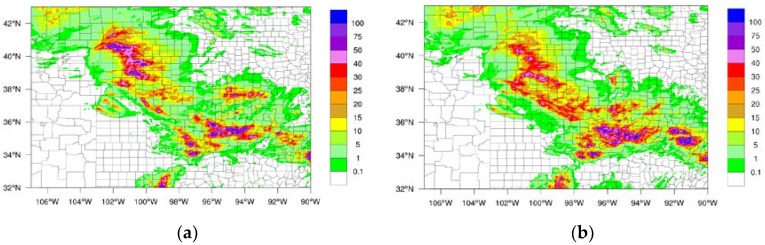
The 18-h accumulated precipitation (unit: mm) from 0000 UTC 24 June to 1800 UTC 24 June, 2018 based on forecast of 3-h latency (**a**) and the forecast of no latency (**b**).

For the LSS Case I, precipitation is one of the most important features for weather forecasting. Figure 3a shows the observed precipitation from Stage IV from 0000 UTC on 24 June to 1800 UTC on 24 June, 2018. The maximum precipitation accumulation was greater than 100 mm during the 18-h period. The rainfall belt occurred from the northwest toward the southeast in the domain area. The simulated precipitation from the 3-h latency experiment is shown in Figure 5a. The main precipitation pattern differed from the observations. The 3-h latency assimilation did not capture the rainfall belt, and the pattern of simulated precipitation from the no latency experiment (Figure 5b) was more similar to the observations than the pattern of simulated precipitation from the 3-h latency (Figure 5a). The rainfall belt from the no latency experiment was observed from the northwest to the southeast. The maximum accumulated precipitation was also over 100 mm during the 18-h forecast period.

**Figure 6:**
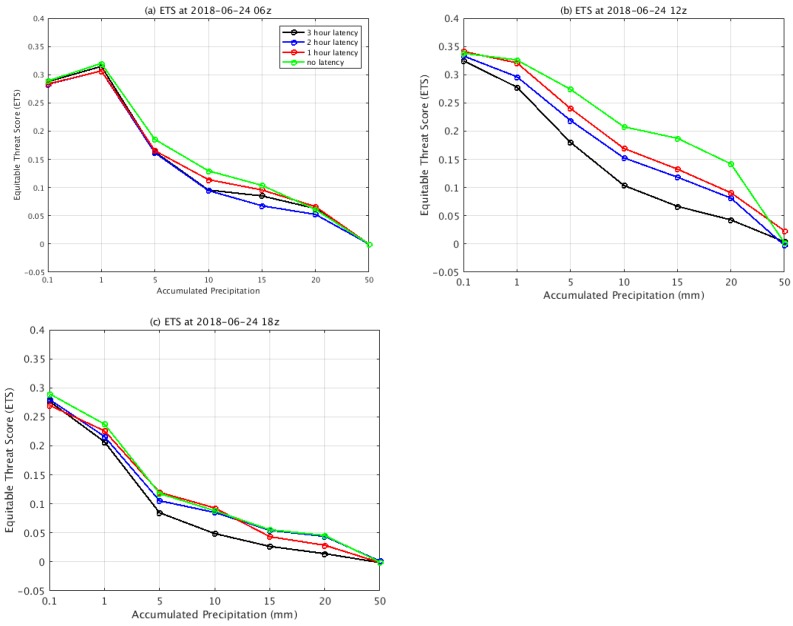
ETS of the 6-h forecasts (**a**), 12-h forecasts (**b**) and 18-h forecasts (**c**) for the experiments of 3-h latency (black), 2-h latency (blue), 1-h latency (red) and no latency (green), respectively.

To further evaluate the impact of latency impacts on the precipitation forecast, the equitable threat score (ETS), probability of detection (POD), and false alarm ratio (FAR) [23,24] scores were calculated for accumulated precipitation at six hour intervals. The resolution of the observed precipitation data was 4 km × 4 km, which was coarser than the 3-km resolution of domain 2. The forecast precipitation was interpolated to the observation grid points and then the ETS, POD, and FAR scores were calculated for a box from 32°N to 43°N latitude and from 90°W to 107°W longitude. During the first 6-h forecast (from 0000 UTC 24 to 0600 UTC 24 June), the ETS of 0.1 mm and 1 mm were similar for all of the experiments (Figure 6). The ETS scores differed among the experiments for accumulated precipitation values over 10 mm. From 0600 UTC on 24 June to 1200 UTC on 24 June, the ETS scores showed significant differences among all of the experiments. The ETS of the no data latency yielded the highest value in comparison to the ETS of the other experiments. In addition, the 3-h latency resulted in the lowest ETS. This indicates that the data latency directly affects the precipitation simulation. When there are data available in the assimilation window, the resulting precipitation forecast is more accurate. The difference in the precipitation forecast was more obvious for the heavy rainfall periods. The ETS scores were similar at 0.1 mm precipitation, but for accumulated precipitation over 5 mm, there were larger differences between the ETS scores. Both the POD and FAR had a similar pattern to the ETS.

### 4.2. Impact on T/Q/U/V Forecasts

**Figure 7:**
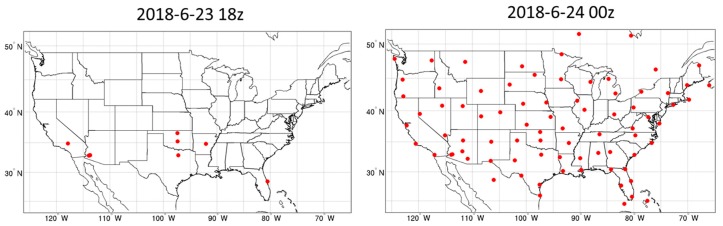
The locations of radiosonde stations at 1800 UTC 23 June and 0000 UTC 24 June, 2018.

In addition to evaluating the precipitation amounts, the forecast fields (T/Q/U/V) were also compared with the radiosonde profiles. The radiosonde stations at 1800 UTC on 23 June and 0000 UTC on 24 June, 2018 are shown in Figure 7 as examples. There were more radiosonde stations available at 1200 UTC and 000 UTC than at 0600 UTC and 1800 UTC. There were a total of 177 radiosonde stations during the forecast time period. Since there are different levels for each observation at each station, the total number of temperature data points was 8245, the number of moisture data points was 6226, and the number of U and V wind data points were 11,433. All observations from the radiosonde stations were compared with the forecast fields at the same time and same level.

The root mean square errors (RMSEs) of the variables (T/Q/U/V) were calculated for all four experiments (Table 3). The smallest RMSEs of each variable among the four experiments are shown in red. The smallest RMSEs of temperature and U-wind were derived from the 1-h latency, and the smallest RMSEs of moisture and V-wind were derived from no latency, indicating that the forecast fields of 1-h latency and no latency were the least affected by the observations. The large data latency of the 3-h and 2-h latency conditions provided less data used for assimilation into the GSI system, which makes the forecast fields worse than the results of 1-h latency and no latency. The results of forecast fields (temperature (T), moisture (Q), and winds (U/V)) were consistent with the precipitation forecasts that more data or low data latency can provide better overall forecast results.

### 4.3. Overall Impact

Instead of showing the results from individual atmospheric fields, an overall evaluation strategy was carried out. The purpose of this strategy was to use one single parameter to characterize the overall impact for the LSS simulations. The RMSEs of temperature (T), moisture (Q), and winds (U/V) compared with radiosondes (Table 3) were used for the calculation. In addition, the precipitation scores (ETS/POD/FAR) were also calculated for the final single parameter calculation. To be consistent with the RMSEs from T/Q/U/V where the lower values are better results, (1-ETS) and (1-POD), the differences between one, and ETS/POD scores of 0.1 mm as the threshold are used. When combining the different units for each parameter, a normalization process was used to ensure the sum of the square equals 1.0 for each parameter.

Table 4 shows the normalized RMSE for all parameters for LSS Case I. Table 5 shows the normalized RMSE using the same method for LSS Case II. The final nominalized RMSE was calculated using a weighted average based on the approach used in the geostationary advanced IR sounder [25] and the CubeSat sounder [26] impact studies:

- Temperature (T), 10%
- Moisture (Q), 10%
- U-wind (U), 10%
- V-wind (V), 10%
- 1-ETS, 20%
- 1-POD, 20%
- FAR, 20%

The precipitation scores were given relatively large weights because of their importance in the LSS simulation. Atmospheric fields were calculated at the last analysis time (1800 UTC 23 June, 2018) and for every 6-h forecast (0000 UTC, 0600 UTC, 1200 UTC and 1800 UTC 24 June) for Case I. The atmospheric fields were calculated at the last analysis time (0000 UTC 24 June 2018) and for every 6-h forecast (0600 UTC, 1200 UTC, 1800 UTC 24 June, 0000 UTC, 0600 UTC, and 1200 UTC June 2018) for Case II. The precipitation scores were calculated for every 6-h accumulated rainfall measurement at 0000 UTC, 0600 UTC, 1200 UTC, and 1800 UTC on 24 June for Case I, and at 0000 UTC, 0600 UT, and 1200 UTC on 25 June for Case II.

**Figure 8:**
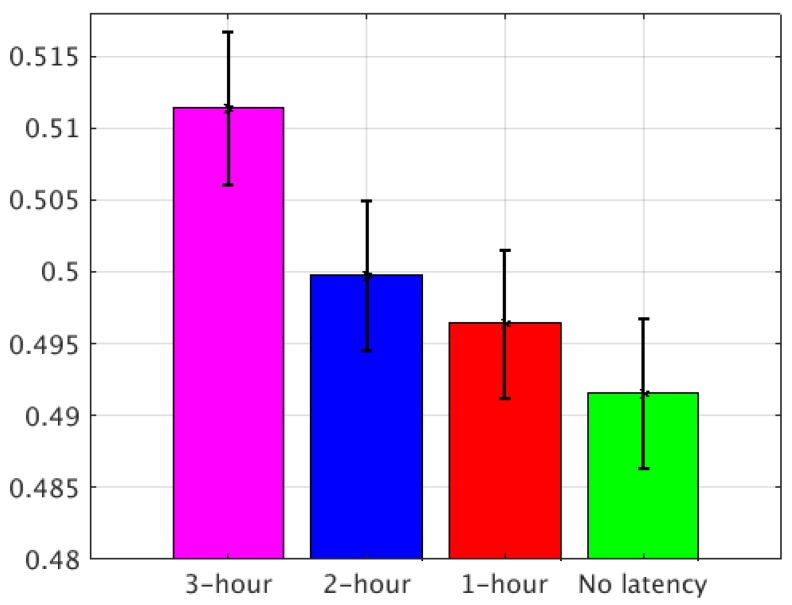
The final normalized RMSE of the four experiments with the 95% confidence intervals for Case I.

**Figure 9:**
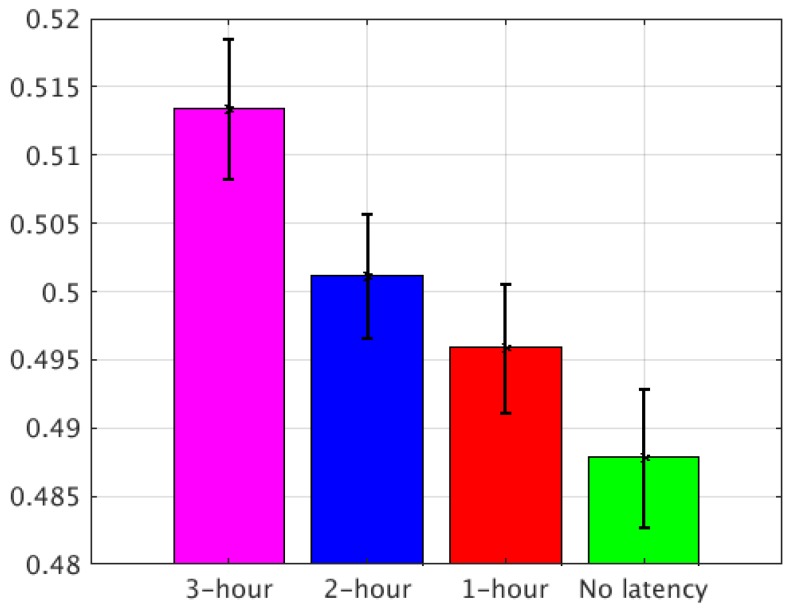
The final normalized RMSE of the four experiments with the 95% confidence intervals for Case II.

Using the method described above, the final normalized RMSEs of the four groups of experiments were calculated with a confidence interval of 95%. For Case I, comparing the four groups of experiments, the improvement from the 3-h latency to the 2-h latency was about 2.3%, from the 3-h latency to the 1-h latency was about 2.9%, and from the 3-h latency to no-latency was about 3.9% (Figure 8). For Case II, the improvement from the 3-h latency to the 2-h latency was about 2.38%, from the 3-h latency to the 1-h latency was about 3.42%, and from the 3-h latency to no-latency was about 4.99% (Figure 9). Based on the two LSS cases, the no-latency experiment provided the most observations to the LSS simulation in the assimilation window for both the analysis and forecast.

## 5. Summary

The forecast of LSS and other rapidly changing weather events depends on how frequently and quickly the forecasts are updated. The low latency sounder data from satellites are very important in improving the pre-convection atmospheric conditions in NWP based, short-range forecasts. Such low latency data from polar orbiting satellites are available from direct broadcast (DB) sites, while the low latency data from geostationary satellites are available from GRB. In this study, the impact of low latency from sounders onboard polar orbiting satellites were demonstrated. Our findings indicate that (1) data latency directly affects the amount of data that can be assimilated into an NWP system and for data coverage, almost all types of satellite observations are increased from 3-h latency to no latency; (2) low latency from polar orbiting satellites results in better forecasts for precipitation and forecast fields (T/Q/U/V) of 1-h latency and no latency are much closer to the observations, and these low latency LEO data can be obtained via DB sites; and (3) the final normalized RMSEs of T/Q/U/V/1-ETS/1-POD/FAR indicate the following order in terms of better impact: no latency > 1-h latency > 2-h latency > 3-h latency. As the no latency condition provides the most observations to the assimilation system, it produced the best results for the LSS forecast.

Two typical LSS cases were demonstrated in this study and the impact of different latencies on regional NWP-based forecasts were clearly distinguished, indicating that the forecasts are sensitive to the amount of sounder data assimilated. The impact, however, is dependent on many other factors including, but not limited to, the parent model used for the initial and boundary conditions for the regional NWP model, the sounder observational characteristics (spatial and temporal resolutions, spectral channels, observation errors, etc.), data assimilation system, and weather situations. It is worth noting that the data assimilation technique is extremely important for low latency data assimilation: more data can be assimilated with a 4DVAR system and a large assimilation window. Additionally, a combination of an advanced satellite data assimilation system and the low latency data could improve high impact weather forecasts. Furthermore, geostationary-based high temporal and spatial resolution observations or derived products such as moisture and dynamic information are very useful for improving tropical cyclone forecasts, especially the rapid scan based mesoscale atmospheric motion vectors (AMVs) in the inner core region. Such information can be obtained via the GRB system. It should be noted that GEO data from GRB have almost no latency; however, the processing time for deriving the products (e.g., AMVs) becomes important for timely assimilation for rapid update of the regional NWP model. In all circumstances, efficient data processing is critical to ensure low latency from both LEO and GEO for these applications.

The results showed that low latency can lead to an improved and positive impact on precipitation and other forecasts, which further indicates the potential of applying LEO DB data in high regional NWP for LSS forecasts.

## Figures and Tables

**Table 1:** The available data hours and data latency.

Latency Time	Data Hours	Available Time
3-h data latency	3-h data	1500 UTC 23 to 1800 UTC 23 June
2-h data latency	4-h data	1500 UTC 23 to 1900 UTC 23 June
1-h data latency	5-h data	1500 UTC 23 to 2000 UTC 23 June
No data latency	6-h data	1500 UTC 23 to 2100 UTC 23 June

**Table 2:** The data coverage increasing rate at 1800 UTC 23 June, 2018.

Satellite	3-h Latency	2-h Latency	1-h Latency	No Latency	Rate
AMSU-A N15 Ch6	142	142	142	142	----
AMSU-A N18 Ch6	1466	1466	1466	1466	----
AMSU-A N19 Ch6	0	107	107	120	**100%**
AMSU-A MetopA Ch6	2179	2182	2181	2182	----
AMSU-A MetopB Ch8	2365	2624	2624	2624	**9.9%**
ATMS Ch11	508	508	1077	1077	**52.8%**
CrIS Ch96	1231	1231	2686	2686	**54.2%**
IASI MetopA Ch110	806	806	806	806	----
IASI MetopB Ch110	1445	1594	1594	1594	**9.3%**

**Table 3:** The RMSE of the T/Q/U/V between the radiosonde profiles and the forecast fields of the four experiments.

RMSE	3-h Latency	2-h Latency	1-h Latency	No Latency
T (K)	1.3999	1.3839	1.3824	1.3979
Q (%)	1.4219	1.4016	1.3902	1.3773
U (m/s)	3.4746	3.4808	3.4420	3.4737
V (m/s)	3.6657	3.6878	3.6541	3.6438

**Table 4:** The normalized RMSE (The Root Mean Square Errors) for T/Q/U/V and precipitation scores for the four experiments of Case I.

RMSE	3-h Latency	2-h Latency	1-h Latency	No Latency
T	0.5029	0.4975	0.4970	0.5025
Q	0.5086	0.5013	0.4973	0.4926
U	0.5010	0.5019	0.4963	0.5009
V	0.5004	0.5034	0.4988	0.4974
1-ETS 0.1 mm	0.5024	0.5007	0.5011	0.4958
1-POD 0.1 mm	0.5493	0.4951	0.4835	0.4683
FAR 0.1 mm	0.4989	0.5011	0.5027	0.4973

**Table 5:** The normalized RMSE for T/Q/U/V and precipitation scores for the four experiments of Case II.

RMSE	3-h Latency	2-h Latency	1-h Latency	No Latency
T	0.5341	0.4928	0.4574	0.5125
Q	0.5389	0.4927	0.4809	0.4852
U	0.5512	0.4993	0.4682	0.4772
V	0.5375	0.496	0.4764	0.4881
1-ETS 0.1 mm	0.5027	0.5048	0.5055	0.4867
1-POD 0.1 mm	0.4765	0.5002	0.5239	0.4983
FAR 0.1 mm	0.5071	0.5106	0.5086	0.4726
